# Sesquiterpene lactone! a promising antioxidant, anticancer and moderate antinociceptive agent from *Artemisia macrocephala* jacquem

**DOI:** 10.1186/s12906-016-1517-y

**Published:** 2017-01-07

**Authors:** Mohammad Shoaib, Ismail Shah, Niaz Ali, Achyut Adhikari, Muhammad Nawaz Tahir, Syed Wadood Ali Shah, Saiqa Ishtiaq, Jahangir Khan, Shahzeb Khan, Mohammad Naveed Umer

**Affiliations:** 1 Department of Pharmacy, University of Malakand Chakdara, Dir Lower, KPK Pakistan; 2 Institute of Basic Medical Sciences, Khyber Medical University, Peshawar, KPK Pakistan; 3 International Center for Chemical and Biological Sciences, H. E. J. Research Institute of Chemistry, University of Karachi, Karachi, Pakistan; 4 Department of Physics, University of Sargodha, Sarghodah Punjab, Pakistan; 5 University College of Pharmacy, University of the Punjab, Punjab, Pakistan; 6 Department of Chemistry, University of Malakand Chakdara, Dir Lower, KPK Pakistan

**Keywords:** *Artemisia macrocephala*, Sesquiterpene lactone, Anticancer, 3 T3, HeLa, MCF-7, DPPH, ABTS

## Abstract

**Background:**

Sesquiterpene lactones (STLs) make a diverse and huge group of bio-active constituents that have been isolated from several plant families. However, the greatest numbers are present in Asteraceae family having more than 3000 different reported structures. Recently several researchers have reported that STLs have significant antioxidant and anticancer potentials.

**Methods:**

To investigate the antioxidant, anticancer and antinociceptive potentials of STLs, gravity column chromatography technique was used for isolation from the biologically rich chloroform fraction of *Artemisia macrocephala* Jacquem. The antioxidant activity of the isolated STLs was determined by DPPH and ABTS free radical scavenging activity, anticancer activity was determined on 3 T3, HeLa and MCF-7 cells by MTT assay while the antinociceptive activity was determined through acetic acid induced writhings, tail immersion method and formalin induced nociception method.

**Results:**

The results showed that the STLs of *Artemisia macrocephala* possesses promising antioxidant activity and also it decreased the viability of 3 T3, HeLa and MCF-7 cells and mild to moderate antinociceptive activity.

**Conclusion:**

Sesquiterpenes lactones (STLs) are widely present in numerous genera of the family Asteraceae (compositae). They are described as the active constituents used in traditional medicine for the treatment of various diseases. The present study reveals the significant potentials of STL and may be used as an alternative for the management of cancer. Anyhow, the isolated compound is having no prominent antinociceptive potentials.

**Electronic supplementary material:**

The online version of this article (doi:10.1186/s12906-016-1517-y) contains supplementary material, which is available to authorized users.

## Background

Sesquiterpenoids are extensively dispersed in plant kingdom. They are natural products with 15 carbons [1–3]. Numerous of them show cyclic chemical structures [4–10]. These compounds are synthesized by plants in several organs such as leaves, fruits or roots [11–14].

Sesquiterpene lactones (STLs) are a sub-class of sesquiterpenoids and are typical secondary compounds of the Asteraceae plant family [15, 16]. In the last several years, the potent bioactivities of STLs, such as anti-malarial activity of artemisinin [17], drew significant interests in the biochemistry of STLs [18]. They show anti-microbial activities, serve as antifeedants [19–21], as an antimigraine [22, 23], anti-inflammatory, for treatment of stomach-ache, skin infection [24], antitumor, antiulcer, neuro-cytotoxic, cardiotonic activities [25] and antineoplastic activity and hence make lead structures for the development of therapeutic agents. According to scientific evidence, the lactone fraction obtained from *Artemisia annua* L have greater potential for pain relief revealed by chemical-induced nociception assays in mice [26]. Similarly several experiments have shown the antinociceptive potential of terpenes in different painful conditions. They were able to reduce significantly the nociceptive response in various models of nociception with possible involvement of muscarinic, opioid, dopaminergic, adenosinergic and glutamatergic systems and the involvement of ATP-sensitive K^+^ channels [27]. Citronellal, a monoterpene possess antinociceptive potentials with the involvement of opioid system [28, 29], similarly carvacrol, p-cymene, hydroxydihydrocarvone (monoterpene) possess antinociceptive potentials in several models of animals [30–32]. Through structural analysis and the mechanism proposed by scientific studies, the monoterpenes like myrcene, linalool, citronellol and citronellal usually have antinociceptive potentials via acting on the opioid system [33].

In view of these, we studied the Hirshfeld analysis and fingerprint plots of the crystal of STL isolated from *Artemisia macrocephala* Jacquem and subjected to antioxidant and cytotoxic study.

## Methods

### Collection and authentitacation of plant materials

Aerial parts of *Artemisia macrocephala* (*A. macrocephala*) were collected from Badwan chowk, Dir Lower, Khyber Pakhtunkhwa, Pakistan in the month of April, 2015. The plant was identified by Professor Jahandar Shah. A voucher specimen, AM-01-2015, has been submitted in Malakand University herbarium.

### Extraction fractionation and isolation of sesquiterpene lactone

Shade-dried (10 kg) aerial parts of *A. macrocephala* were pulverized. This material was then soaked in commercial grade methanol with occasional stirring. Filtered the whole suspension after 22 days and repeated this process three times. Then combined all the filtrates and concentrated through rotary evaporator under reduced pressure at 40 °C. 1 kg of methanolic greenish-black extract was obtained which was subjected to successive fractionation.

The crude extract was added to 5 l distilled water followed by the addition of an equal volume of *n*-hexane and shaken gently in a separating funnel. Collected the *n*-hexane layer from the separating funnel and continued this process till there appeared no color in the *n*-hexane when added further. Then combined the entire *n*-hexane portion and subjected to rotary evaporator at 45 °C, 120 g *n*-hexane fraction (12%) was collected. The process was repeated for chloroform (37.8%), ethyl acetate (12.2%) and *n*-butanol (15.4%) to obtain their respective fractions. The remaining aqueous portion left was approximately 210 g (21%).

### Chemical characterization of crude extract/fractions and isolation of sesquiterpene lactone

The crude methanolic extract and fraction were tested for the presence of terpenoids [34]. The fraction showing positive result (chloroform fraction) was subjected to column chromatography for the isolation and purification of terpenoids by elution with 5% ethyl acetate: *n*-hexane. It gave the compound Ism-1 (1.3 g). TLC plate was used for examining the purity of the compound and visualized under UV lamp. Further spectral and crystal data lead to the confirmation of the compound as sesquiterpene lactone (STL).

### DPPH free radical scavenging assay

The antioxidant activity of the isolated STL was carried out through its scavenging ability of DPPH free radical. Various dilutions of 25, 50, 75, 100, 125 and 150 μM of the isolated compound were mixed with 1 ml solution of DPPH (90 μM) and adjusted the final volume 4 ml via methanol. After a time period of 1 h the absorbance of test solutions and the blank was recorded at room temperature. Ascorbic acid helped as positive control. Each reading was taken in triplicate. Percent (%) scavenging of DPPH free radical was calculated as.

$$ \%\ \mathrm{Scavenging} = 100 \times \left({\mathrm{A}}_{\mathrm{blank}}\_\ {\mathrm{A}}_{\mathrm{sample}}/\ {\mathrm{A}}_{\mathrm{blank}}\right) $$

Where A_blank_ is absorbance of control, A_sample_ is the absorbance of the test sample.

IC_50_ values, which represented the concentration of test sample that caused 50% neutralization of DPPH radical, were calculated from the plot of inhibition percentage against concentrations [35].

### ABTS free radical scavenging assay

The antioxidant activity of the isolated compound was evaluated against ABTS free radical [36]. This is dependent upon the potential of antioxidants to scavenge ABTS radical cation causing a reduction in absorbance at 734 nm. Shortly, K_2_S_2_O_4_ solution (2.45 mM) was mixed with ABTS (7 mM) solution. Kept this mixture in dark at room temperature for 14 h which gave a dark color solution having cation radical of ABTS. The cation radical solution of ABTS was then diluted with 0.01 M phosphate buffer of pH 7.4 in order to adjust the absorbance 0.70 at 734 nm. ABTS solution (3 ml) was mixed with 300 μl of test sample to determine the radical scavenging potential of the test samples. Reduction in absorbance was determined through spectrophotometer after 1 min of the solutions mixing and continued for 6 min. Ascorbic acid served as positive control. All the readings were taken in triplicate. Percentage scavenging was calculated as:

$$ \%\ \mathrm{scavenging}\ \mathrm{effect}\kern0.5em =\frac{\mathrm{control}\ \mathrm{absorbance}\ \hbox{-}\ \mathrm{sample}\ \mathrm{absorbance}}{\mathrm{control}\ \mathrm{absorbance}}\times 100 $$

Antioxidant potential was expressed as percent inhibition and as IC_50_ (Test sample concentration required for 50% reduction of ABTS radicals).

### Anticancer activity

Anticancer activity of the isolated STL was carried out in 96-well flat-bottomed micro plates through standard MTT (3-[4, 5-dimethylthiazole-2-yl]-2, 5-diphenyl-tetrazolium bromide) colorimetric assay [37]. For this purpose, HeLa (Cervical Cancer), MCF-7 (Breast Cancer) and NIH 3 T3 (Mouse fibroblast) were cultured in Minimum Essential Medium Eagle, supplemented with 5% of fetal bovine serum (FBS), 100 IU/ml of penicillin and 100 μg/ml of streptomycin in 75 cm^2^ flasks, kept at 37 °C in 5% CO_2_ incubator. Exponentially growing cells were harvested, counted with haemocytometer and diluted with a particular medium. Cell culture with the concentration of 6×10^4,^ 5×104 and 5×10^4^ cells/ml for HeLa, MCF-7 and NIH 3 T3 respectively was prepared and introduced (100 μL/well) into 96-well plates. After overnight incubation, medium was removed and 200 μL of fresh medium was added with different concentrations of compound (62.5-1000 μg/mL). After 48 h, 200 μL MTT (0.5 mg/ml) was added to each well and further incubated for 4 h. Subsequently, DMSO (100 μL) was added to each well. The extent of MTT reduction to formazan within cells was calculated by measuring the absorbance at 570 nm, through micro plate reader (Spectra Max plus, Molecular Devices, CA, USA). The cytotoxicity was recorded as concentration causing 50% growth inhibition (IC_50_) for HeLa. Percent inhibition was determined as:

% inhibition = 100-[(mean of O.D of test compound – mean of O.D of negative control)/(mean of O.D of positive control – mean of O.D of negative control) × 100].

The results (% inhibition) were processed by using Soft- Max Pro software (Molecular Device, USA).

#### Acute toxicity

Balb/C mice (18–26gm) were purchased from National Institute of Health (NIH) Islamabad and housed in separated cages at University of Malakand animal house with free access to standard diet and water and starved for 12–18 h before experimentation. Ethical Committee of Pharmacy Department, University of Malakand accepted the protocols (No: S-Ter-24-12/2015) of experimental and ensured its compliance with provisions of the “Animal Bye-Laws 2008, Scientific Procedures Issue-I of the University of Malakand”.

The isolated STL was tested for acute toxicity study as per standard protocol [38]. Balb C mice were given 25, 50 and 100 mg/kg of the isolated compound in phase first. While 125, 250 and 500 mg/kg in phase second were given to the experimental animals. One group, given normal saline served as control. They were observed for 6 h continuously for changes in the behavioral or autonomic responses followed by another examination after 24 h. Any mortality for the next 14 days was also noted.

#### Acetic acid induced writhing test

The peripheral antinociceptive response was determined through acetic acid induced writhing test. Two groups of mice (*n* = 6) for each sample were prepared. They were given 25 and 50 mg/kg of isolated compound 1 h before the injection of 10 ml/kg of 1% acetic acid intraperitoneally. Negative control group was given 10 ml/kg of 1% solution of Tween 80 while positive control group was given 10 mg/kg of diclofenac sodium intraperitoneally to overnight fasting mice. Writhing and stretching number was noted and percent protection was determined from the data [39].

#### Thermal nociception (Tail immersion method)

In order to determine the central antinociceptive response of the sample, the animals were given 25 and 50 mg/kg of isolated compound intraperitoneally, 2% vehicle and 10 mg/kg of diclofenac sodium, 30 min prior to the immersion of the tail (3 cm) into hot water at a temperature of 55 ± 0.5 °C. To assist the possible involvement of opioid receptor, morphine (agonist) at a dose of 5 mg and naloxone (antagonist) at a dose of 2 mg were used. The time of reaction taken at 15, 30, 45, 60, 75 and 90 min after administration of sample were noted with a stopwatch [40].

#### Formalin induced writhings test

This test was carried out with in Balb C mice using formalin. 25 and 50 mg/kg of isolated compound (i.p) were given to the pre labeled groups. 30 min after the treatment of test samples, 2.5% formalin (20 μl) was injected (s.c) in mice hind paw. The time spent in licking the injected paw in early phase (0–5 min) and late phase (15–30 min) was recorded. Naloxone, opioid antagonist (2 mg/kg, s.c) and standard drug indomethacin (10 mg/kg) was also used [41].

#### Statistical analysis

All the experiments were carried out in triplicate. The data was represented as mean ± SEM. Data was subjected to one way ANOVA followed by Dunnetts test for finding statistical significance. P value less than 0.1 was considered statistically significant.

## Results

### Structures of isolated compound

Colorless needle. ^1^H-NMR (CD_3_OD, 400 MHz): δ: 4.81 (dd, 1H, J= 1.0 Hz, J= 11.0 Hz, H-6), 2.64 (m, 1H, H-11), 2.61 (m, 1H, H-2β), 2.47 (m, 1H, H﻿-2α), 2.39 (m, 1H, H-3β), 2.34 (m, 1H, H-3α), 2.17 (m, 1H, H-7), 1.94 (s, 3H, H-15), 1.84 (m, 1H, H-9β), 1.81 (m, 1H, H-8α), 1.66 (m, 1H, H-8β), 1.51 (m, 1H, H-9α), 1.30 (s, 3H, H-14), 1.19 (d, 3H, J 7.5 Hz, H-13). EI-MS (*m/z*) 248 [M] ^+^ [42].

**Fig. 1:**
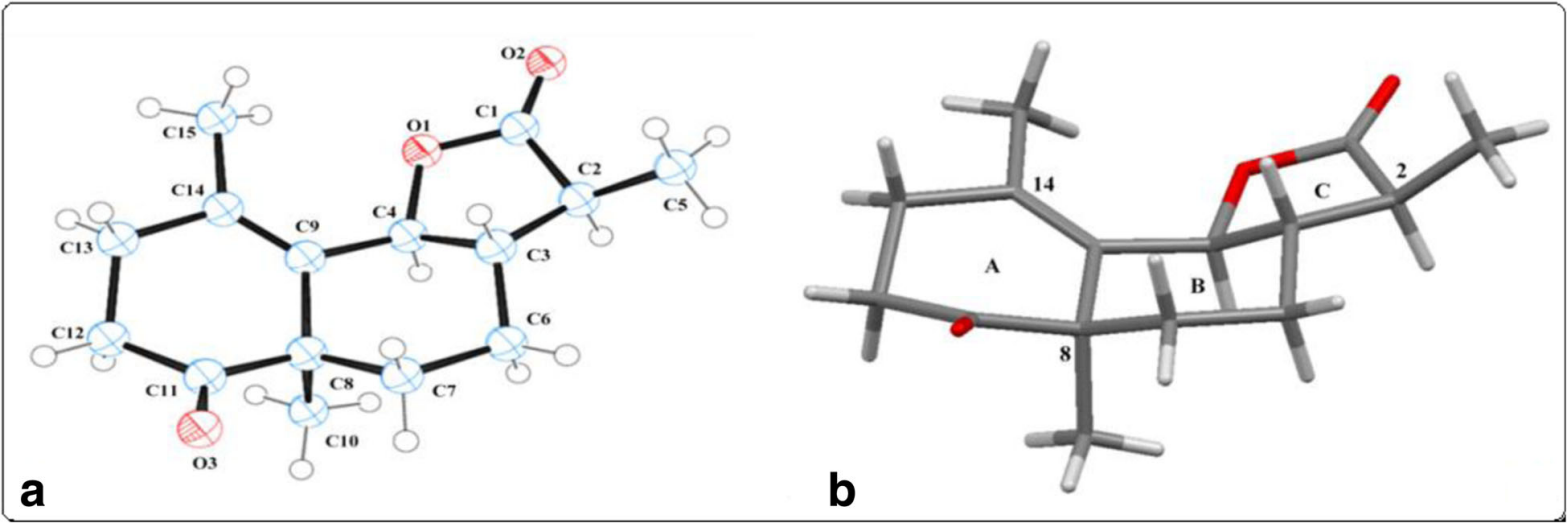
**a** ORTEP view of the title molecule with 50% probability, **b** conformation of rings and orientations of methyl substitutions

The title compound (C_15_H_20_O_3_), comprises on one hexahydroxynaphthol ring (C3/C4/C6-C9/C11-C14), having a ketonic functionality at C-11 and dimetyl substitutions at C-8 and C-15 along with five membered cyclic ester [lactone, C1-C4/O1/O2 (Fig. 1a)].

Ring A adopts a half-chair conformation while ring B adopts complete chair conformation and ring C possess enveloped conformations (Fig. 1b). The title compound has three methyl substitutions, one methyl group is located on fused C-8, the second methyl group is located on C-14 and both has axial orientations. The third methyl group has pseudo-axial orientation on C-2 make the dihedral angle to the lactone ring (C-C2-C5) is 112.7 (3) °. The torsion angle of the plane (C8-C9-C14-C13) of ring A is 1.4 (6)°.

**Fig. 2:**
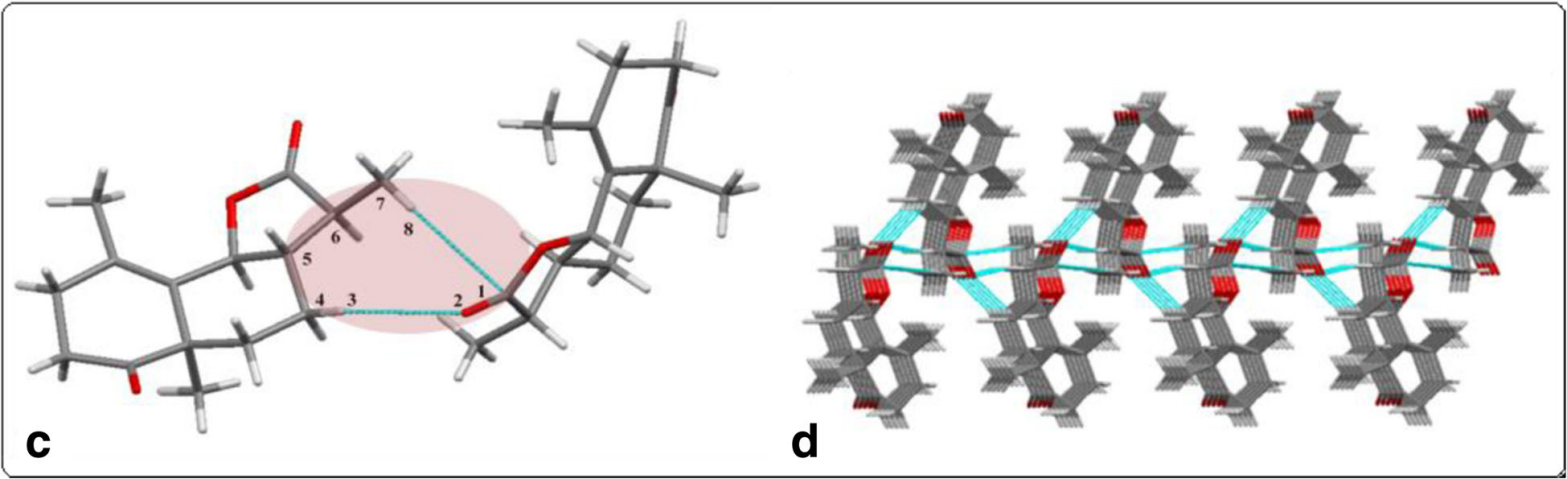
**c** Formation of dimer via C-H•••O and C-H•••C intermolecular interactions, **d** molecular packing of compound like zig-zag pattern along *a*-axis

In the crystal, asymmetric molecule is directly interlinked with other four neighboring molecules through intermolecular interactions. Molecules are arranged in the crystal packing in such a way that making two dimer motifs via C-H•••O (C6-H6A•••O2 = 153.8° and H6A•••O2 = 2.709 Ǻ) and C-H•••C (C5-H5A•••C1 = 164.9° and H5A•••C1 = 2.892 Ǻ) intermolecular hydrogen bonding (Fig. 2c). Additionally, the O•••H (O2•••H2 = 2.507Ǻ) intermolecular interactions are involved in the formation of zig-zag anti-parallel chain in a two-dimensional network (Fig. 2d).

**Fig. 3:**
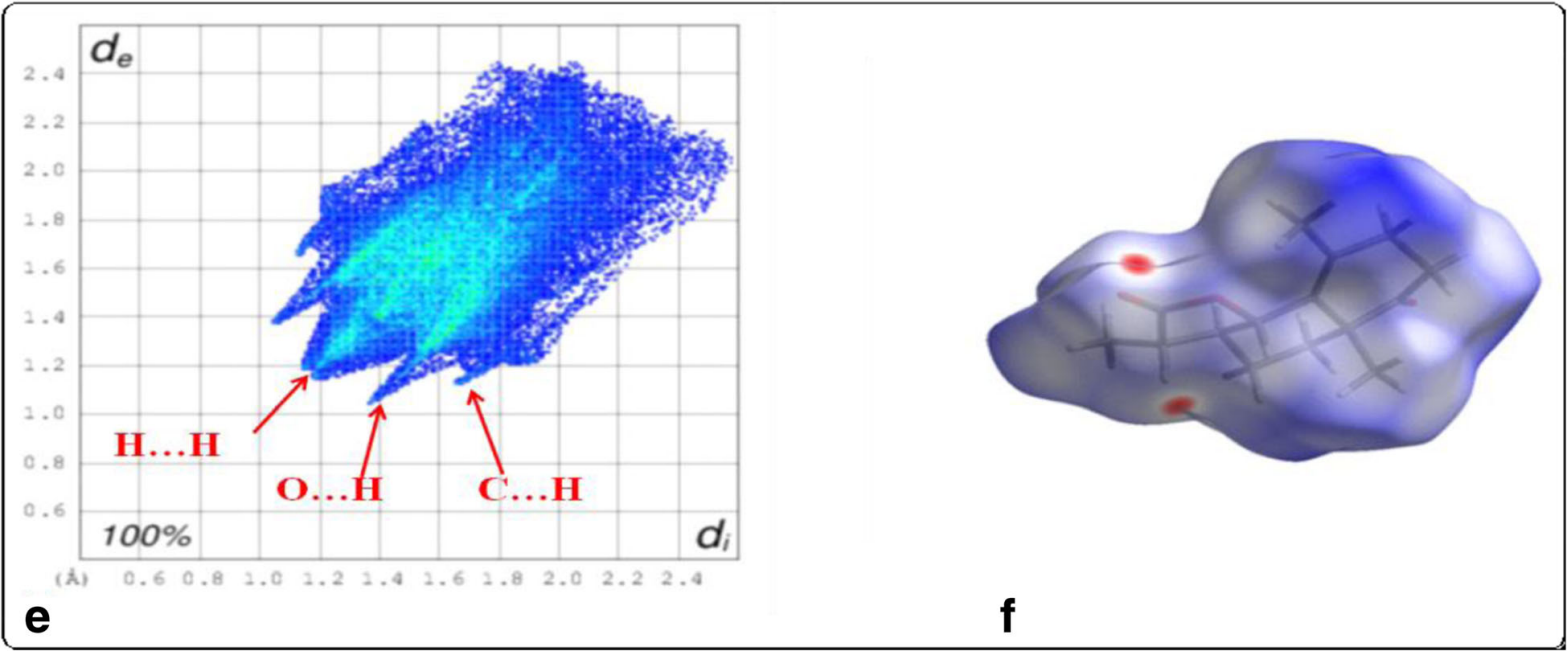
**e** Hirshfeld surface fingerprint plot, and (**f**) the d_norm_ surface of title molecule

**Fig. 4:**
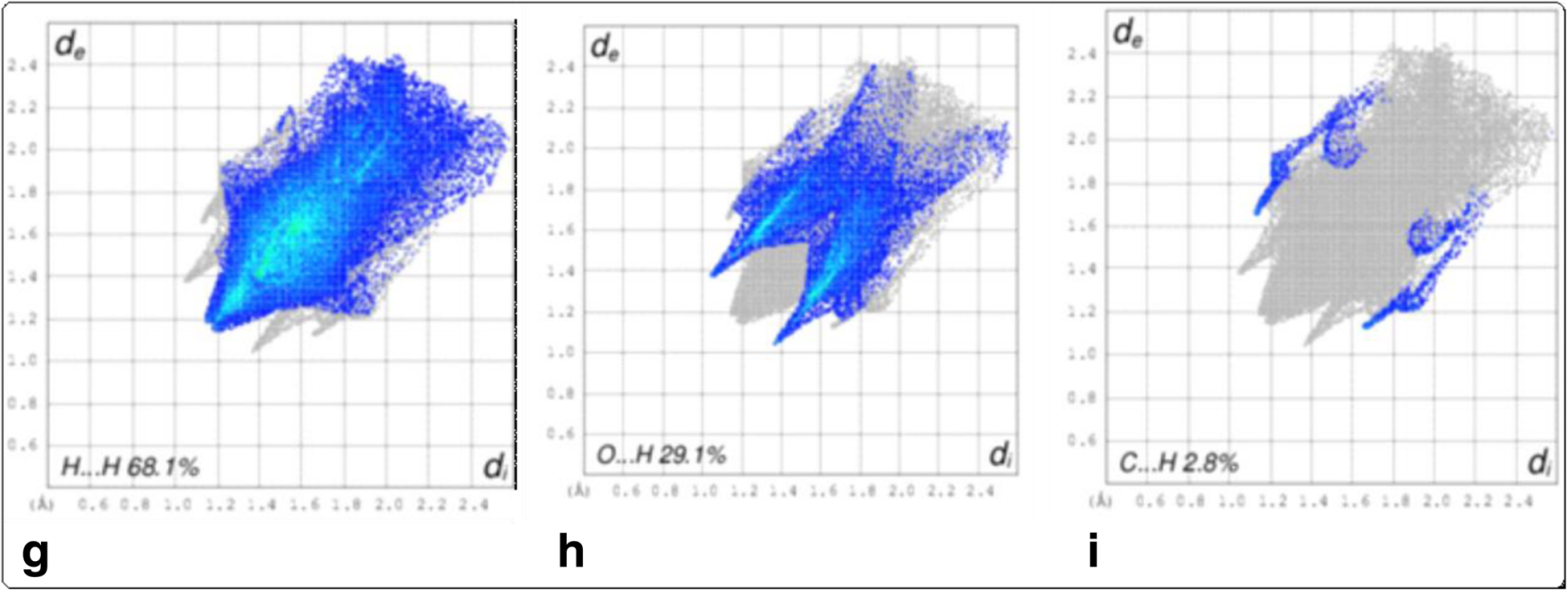
**g** The proportion of H•••H, (**h**) O•••H, and (**i**) C•••H contacts in title molecule

### Hirshfeld surface and fingerprint plots analysis

**Fig. 5:**
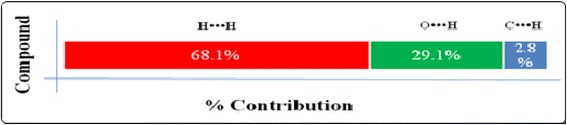
Percentage contributions to the Hirshfeld surface areas for the various intermolecular contacts (H/H, O/H and C/H) in molecule

Hirshfeld surface analysis is a powerful tool in the estimation and understanding the nature of intermolecular interactions involving in the packing of molecular crystal by using their fingerprint plots. In the title molecule, the Hirshfeld surface analysis shows a higher proportion of H•••H contacts, ranging from 60 to 70% as compared to O•••H, C•••H contacts (Fig. 3). In this case, the H•••H (Fig. 4) interactions are represented by the mid area of the fingerprint plots, which represents an asterisk oxygen interacting with the neighboring α-hydrogen of location, forming the zig-zag network of hydrogen bonds (Fig. 5).

The sharp “wings” seen in the plot belong to C-H•••O interactions, with the “wings” in the lower area of the fingerprint plot representing C-H•••O acceptor (H•••O) interactions. The proportion of O•••H interactions has a larger contribution than its H•••O counterparts. The proportion of O•••H interactions vary from 10.0 to 13.4%, is mainly due to neighboring ester group pointing toward the oxygen of the lactone moiety whereas proportion of O•••H interactions ranging from 12.0 to 15.7%.

### Antioxidant activity of the STL towards DPPH and ABTS free radical scavenging radicals

This method of stable free radical DPPH is normally used to investigate the anti-oxidant potentials. Here the test was conducted to determine the IC_50_ value of STL to scavenge DPPH radicals. The isolated compound shows a dose dependent activity starting from 26.65 ± 1.04 at the lowest concentration to 68.76 ± 0.82 at the highest concentration used. The value is reaching to that of ascorbic acid at the same concentration used as standard. Other standards, tocopherol and rutin are anyhow having higher values at the same concentration of 150 $\mu$g/mL Table 1.

**Table 1:** Percent DPPH radical scavenging activity of STL

	25 $\mu$g/mL	50 $\mu$g/mL	75 $\mu$g/mL	100 $\mu$g/mL	125 $\mu$g/mL	150 $\mu$g/mL
Ascorbic acid	67.33 ± 0.41	73 ± 0.61	77.26 ± 0.68	83.09 ± 0.42	87.11 ± 0.87	88.68 ± 0.58
Tocopherol	71.32 ± 0.36	78.16 ± 0.57	85.41 ± 0.61	91.18 ± 0.53	93.24 ± 0.75	94.33 ± 0.49
Rutin	78.03 ± 0.76	84.11 ± 0.43	86.63 ± 0.81	92.25 ± 0.61	94.35 ± 0.66	96.67 ± 0.51
STL	26.65 ± 1.04	31.62 ± 0.67	40.94 ± 0.63	47.63 ± 0.39	54.74 ± 0.86	68.76 ± 0.82

In the ABTS assay, the STL again showed an activity of 71.76 ± 0.82 at 150 $\mu$g/mL, having an excellent activity reaching to that of ascorbic acid again where as the other standards tocoperol and rutin are having the reading of 94.33 ± 0.49 and 94.67 ± 0.51 at the same concentration Table 2.

**Table 2:** Percent ABTS radical scavenging activity of STL

	25 $\mu$g/mL	50 $\mu$g/mL	75 $\mu$g/mL	100 $\mu$g/mL	125 $\mu$g/mL	150 $\mu$g/mL
Ascorbic acid	70.23 ± 0.41	77.56 0.61	80.26 ± 0.68	85.09 ± 0.42	87.61 ± 0.87	89.88 ± 0.58
Tocopherol	74.32 ± 0.36	78.16 ± 0.57	86.41 ± 0.61	91.18 ± 0.53	93.84 ± 0.75	94.33 ± 0.49
Rutin	79.03 ± 0.76	85.11 ± 0.43	87.63 ± 0.81	89.25 ± 0.61	92.35 ± 0.66	94.67 ± 0.51
STL	30.65 ± 1.04	33.62 ± 0.67	42.94 ± 0.63	45.63 ± 0.39	61.74 ± 0.86	71.76 ± 0.82

### Inhibitory effects of the STL towards the proliferation of 3 T3, HeLa and MCF-7 cells

The sesquiterpenoids have prominent antitumor [25] and antineoplastic activity [43]. To evaluate such potentials of the isolated STL, we investigated its effects on 3 T3, HeLa and MCF-7 cells. The anti-proliferative potentials of the tested STL are expressed in terms of % cells growth inhibition and are depicted in Table 3. The isolated compound was found to be very active against HeLa MCF-7 cells. It showed 64.39 ± 2.40 and 81.25 ± 3.07% cell growth inhibitions against HeLa cell line at 500 and 1000 μg/mL concentrations respectively. Similarly, it revealed 65.34 ± 2.00 and 73.41 ± 2.11% cell growth inhibition against MCF-7 cells at 500 and 1000 μg/mL concentrations respectively. The compound mostly showed no anti-proliferative activity against 3 T3 cells even at the highest concentration of 1000 μg/mL (43.11 ± 1.34%).

**Table 3:** In-Vitro anti-proliferative activity (% cell growth inhibition) of STL against 3 T3, HeLa and MCF-7 cell lines

Sample	Concentration (μg/mL)	3 T3	HeLa	MCF-7
STL	1000	43.11 ± 1.34	81.25 ± 3.07	73.41 ± 2.11
	500	28.25 ± 2.00	64.39 ± 2.40	65.34 ± 2.00
	250	21.91 ± 1.05	53.56 ± 2.00	49.62 ± 2.32
	125	15.00 ± 0.52	37.58 ± 1.78	35.50 ± 1.00
	62.5	09.25 ± 1.56	24.67 ± 1.65	27.50 ± 0.85

#### Acute toxicity

According to International regulations concerning human health oblige that all novel pharmaceutical drugs are tested for safety before use in human beings. An important phase in ensuring drug safety is toxicity tests conduction in proper animal models. The studies of acute toxicity are among the series of toxicity tests used. The aim of this test is the identification of a dose that cause serious adverse effects and evaluation of lowest dose that cause lethality. The International Conference on Harmonization in its 3^rd^ meeting (ICH M3) suggests the acute toxicity studies or appropriate alternatives are essential before administering new medicine in humans for the first time [44].

The isolated STL at a highest dose of 500 mg/kg body weight showed no adverse effect on the behavioral responses in the tested mice for 14 days observation. No mortality or weight change observed. The mice were sacrificed for checking the gross anatomy of liver and kidney, anyhow, no significant changes were observed when compared to normal. Therefore, the dose considered to be safe is 50 mg/kg.

#### Acetic acid induced writhing

The acetic acid test is implicated for the determination of peripheral activity. In performing the acetic acid induced writhing test for the determination of antinociceptive affect, the sample showed a moderate antinociceptive effect. The sample mild to moderately inhibited the acetic acid induced writings with a maximum value of 53.62% (*P* < 0.01, *n* = 6) at 50 mg/kg as shown in Table 4. All the results were compared to the standard (diclofenac sodium, 10 mg/kg) with 83.29% response.

**Table 4:** Acetic acid induced writhing response of pure compounds

Treatment/Dose	Number of writhing	% inhibition
Control (2% Tween 80)	69.75 ± 1.45	---
Diclofenac sodium (10 mg)	11.65 ± 2.02	83.29
Ism-1 25 mg 50 mgs	54.66 ± 1.20^*^	21.63
	32.35 ± 1.35^**^	53.62

All the values were expressed as mean ± SEM (*n* = 6). ^*^ *P* < 0.1, ^**^ *P* < 0.01 when compared to control group (one way ANOVA followed by Dunnetts: compare all *vs* control test)

#### Formalin test

Formalin test is used for continuous moderate pain. It is a valid model for pain determination in animal model. After the administration of the sample to the animals treated with formalin, it again mildly inhibited the first phase with 45.16% (^**^ *P* < 0.01, *n* = 6) and the second phase with 51.47% (^**^ *P* < 0.01, *n* = 6) at 50 mg/kg.

Animals treated with morphine (5 mg) inhibited significantly both the phases with 86.48% (^***^ *P* < 0.001, *n* = 6) and 95.82% (^***^ *P* < 0.001, *n* = 6) for first phase and second phase respectively as shown in Table 5. Indomethacin (10 mg/kg) mildly inhibited the first phase with 27.69% (^***^ *P* < 0.001, *n* = 6) and significantly inhibited the second phase with 73.47% (^***^ *P* < 0.001, *n* = 6).

**Table 5:** Formalin-induced paw-licking response of pure compounds

Treatment/Dose	Licking time (Sec)		Inhibition (%)	
	1st Phase	2nd Phase	1st Phase	2nd Phase
Control (2% Tween 80)	51.85 ± 1.83	73.05 ± 1.75	----	----
Morphine 5 mg	7.01 ± 1.153^***^	3.05 ± 1.290^***^	86.48	95.82
Indomethacin 10 mg	37.35 ± 1.37^**^	19.38 ± 1.440^***^	27.69	73.47
Ism-1 25 mg 50 mg	32.45 ± 1.231^*^	41.12 ± 1.204^*^	37.41	43.70
	28.43 ± 1.356^**^	35.45 ± 1.198^**^	45.16	51.47

All the values were expressed as mean ± SEM (*n* = 6). ^*^ *P* < 0.1, ^**^ *P* < 0.01 and ^***^ *P* < 0.001 when compared to control group (one way ANOVA followed by Dunnetts: compare all *vs* control test)

#### Thermal nociception (tail immersion) test

This test is performed for finding out the central analgesic response. The samples when tested for its effectiveness in tail flick model, significantly increased the latency time at both the doses to 60.00% (^**^ *P* < 0.01, *n* = 6) and 57.89% (^**^ *P* < 0.01, *n* = 6) respectively at 25 mg/kg and 62.10% and 61.05% at 60 min at which morphine (opioid analgesic, centrally acting), showed 85.07% (^***^ *P* < 0.001, *n* = 6) activity. Naloxone treated animals significantly reduced the analgesic potentials of morphine and the isolated compounds (Table 6).

**Table 6:** Tail flick response of isolated compounds

Treatment/Dose	Time in Sec (Tail Flick)/Response (%)					
	15 min	30 min	45 min	60 min	75 min	90 min
Control (2% Tween 80)	0.79 ± 0.017	0.87 ± 0.033	0.99 ± 0.024	0.94 ± 0.036	0.87 ± 0.029	0.90 ± 0.048
Ism-1 25 mg 50 mg	0.95 ± 1.12* (20.25%)	1.07 ± 0.112* (22.98%)	1.30 ± 0.131** (31.31%)	1.33 ± 0.124** (41.48%)	1.33 ± 0.163*** (52.87%)	1.33 ± 0.138*** (47.77%)
	0.98 ± 1.20 (24.05%)	1.11 ± 0.411* (27.58%)	1.34 ± 0.161** (35.35%)	1.37 ± 0.201** (45.74%)	1.38 ± 0.206*** (58.62%)	1.36 ± 0.218*** (51.11%)
Standard (M)	1.20 ± 0.024 (48.14%)	2.03 ± 0.066 (56.15%)	3.89 ± 0.038 (75.06%)	6.30 ± 0.054 (85.07%)	4.40 ± 0.050 (80.22%)	4.30 ± 0.074 (78.37%)
N + 1 25 mg 50 mg	0.93 ± 0.045	0.94 ± 0.043	1.10 ± 0.052	0.99 ± 0.052	0.90 ± 0.058	0.93 ± 0.096
	0.89 ± 0.039	0.95 ± 0.068	1.50 ± 0.064	0.99 ± 0.047	0.89 ± 0.080	0.95 ± 0.089
M + N	0.80 ± 0.046	0.87 ± 0.037	0.94 ± 0.045	0.91 ± 0.033	0.91 ± 0.034	0.91 ± 0.045

All the values were expressed as mean ± SEM (*n* = 6). ^*^ *P* < 0.05, ^**^ *P* < 0.01 and ^***^ *P* < 0.001 when compared to control group (one way ANOVA followed by Dunnetts: compared all *vs* control test)

Key: *M* Morphine 5 mg, *N* Naloxone 2 mg

## Discussion

STLs are pharmacologically active molecules and have been reported for various potentials like antitumor, anti-inflammatory, antibacterial, antifungal, antiviral, antiprotozoal, antihelminthic, antiulcer, molluscicidal, hepatoprotective, hepatocurative, and antidepressant effects [45–65]. The first-line therapy against malaria caused by *Plasmodium falciparum* is, as recommended by the World Health Organization (WHO), a combination therapy of a STL (artemisinin) and its derivatives with other antimalarials, such as mefloquine and amodiaquine [66, 67]. Semisynthetic derivatives with improved pharmacokinetic profiles include the active principle dihydroartemisinin, artemether, artelinic acid and artesunate [66]. Free radicals production in the living systems leads to a series of chemical reactions and thus gives rise to serious tissues injuries and ultimately cancer. STLs have been reported to be effective in inhibiting free radicals [68]. Data from the literature show that some species of the genera Artemisia possess analgesic activity and these pharmacological effects have been attributed mainly to flavonoids, alkaloids, sesquiterpene lactones and essential oils [69]. Currently, STLs have been the subject of greater scientific interest due to their ever-increasing evidence concerning their antitumor properties.

The cytotoxic and apoptotic effects of STLs have been investigated in vitro against several cancer cell lines [70–75]. In result of all the chemical and pharmacological research on STLs, substances such as parthenolide and its synthetic analog, dimethylaminoparthenolide (DMAPT), thapsigargin, the artemisinin derivatives artemether and artesunate are presently in cancer clinical trials [56]. Similarly thapsigargin pro-drug (G-202, thapsigargin coupled with a masking peptide which is cleaved at the tumor site, releasing the cytotoxic drug) is in phase I clinical trials for advanced solid tumors. These substances exert diverse mechanisms of antitumoral action, such as ROS formation, epigenetic modulation of gene expression, targeting the sarco/endoplasmic reticulum calcium ATPase (SERCA) pump, the NF-kB signaling pathway, the p53 pathway, and inhibiting angiogenesis and metastasis [76, 77]. Most recently, Vernolide from *Vernonia cinerea*, has been reported for immunostimulatory effects, inducing enhanced cellular and humoral responses against tumors [78]. Our STL showed good inhibition of gowning cancer cell (HeLa and MCF-7 in case of this study). Although it did not show good results against 3 T3 cell line, may be due the cell line nature that is not enough sensitive like the other two. The possible mechanism for the anti-proliferative activity of the tested STL may be its ability to simultaneously target two molecular pathways (NF-kB and p53) as reported for other STLs elsewhere in literature [79]. Thus, the results of the present study reveal the possible potentials of the isolated STL for its anti-proliferative activity.

The scavenging of free radicals has been important as it helps in preventing the tissues and other vital organs injuries. The isolated STL was screened for free radicals scavenging potentials using DPPH and ABTS free radicals. It caused the scavenging of both the free radicals in a concentration dependent manner. It was found quite effective against the tested free radicals. The isolated STL will require to be extensively studied for its antioxidant activity possible mechanism but mostly STLs have been reported to exert their antioxidant activity through the activation of antioxidant response element (ARE) gene [68]. The results of the present study suggest the possible uses of the isolated STL in free radicals caused tissues injuries.

antinociceptiveantinociceptive. Three different models, visceral nociception, inflammatory nociception and thermal and neurogenic nociception, were used to investigate the antinociceptive effects of the STL. Abdominal constriction assay induced with acetic acid [80] is considered to be sensitive using minimum quantity of noxious stimulus and the results can detect even a weak analgesic. This method is used for investigation of peripheral analgesic response. The acetic acid increases prostaglandin level (especially PGE2) in the mice peritoneal fluid [81]. Prostaglandins activate abdominal constriction via sensitizing and activating peripheral chemo-sensitive nociceptors [82] which are mainly involved in inflammatory pain [83].

Nociception induced with formalin is used to measure the potentials of a substance to relieve continuous moderate pain generated as a result of tissue injury [84]. Formalin induced acute and chronic phases of nociception are regarded to indicate neurogenic and inflammatory pain behaviors respectively. The acute phase is due to direct chemical stimulation of nociceptive afferent fibers (mostly C fibers) which can be suppressed by opiate like morphine [85]. The 2^nd^ phase is due to release of inflammatory mediators such as histamine, prostaglandins, bradykinin, serotonin in the peripheral tissues [85], and from functional changes in the spinal dorsal horn [86]. The tail immersion test is used to determine the spinal pathways in regulation of pain response [87]. This method utilizes elevated thermal nociception and test samples showing good antinociceptive results in this way are regarded potent analgesics [86].

In tail immersion test, a little increase in reaction time after treatment with the sample indicates that the isolated compound is having moderate efficacy in a model of thermal nociception. antinociceptiveThe nociceptive patterns after formalin injection were particularly recorded in two phases. In first phase, paw licking/biting response starts immediately after injection and this may be considered due to direct stimulation of nociceptors [88]. Second phase appears little later and is regarded to be due to combination of inflammatory reactions in the peripheral tissue and changes in central processing’s [89]. A moderate increase in the antinociceptive effect was observed against both neurogenic (early phase) and inflammatory (late phase) pain behavior caused by formalin. The magnitude of inhibition in both the phases was moderate for the isolated compound. It may be suggested from the results that the isolated compound have no prominent antinociceptive potentials.

## Conclusion

Sesquiterpenes lactones (STLs) have been isolated from numerous genera of the family Asteraceae (compositae) and can also be found in other angiosperm families. They are described as the active constituents of a variety of medicinal plants used in traditional medicine for the treatment of various diseases. They are known to possess wide variety of biological and pharmacological activities such as antimicrobial, cytotoxic, anti-inflammatory, antiviral, antibacterial, antifungal activities, effects on the central nervous and cardiovascular systems as well as allergenic potency. Their wide structural diversity and potential biological activities have made further interest in the field of Pharmacy and Pharmacology. The present study was designed to isolate the STLs from *A. macrocephala* and to investigate their possible antioxidant, anticancer and antinociceptive potentials. The study reveals the significant potentials of STL and may be used as an alternative for the management of cancer. Anyhow, the isolated compound is having no prominent antinociceptive potentials.

## Additional file

Additional file 1: NMR spectra of compound. (DOCX 696 kb)
