# Supplementary material for: Sesquiterpene lactone! a promising antioxidant, anticancer and moderate antinociceptive agent from Artemisia macrocephala jacquem
Source: BMC Complement Altern Med. 2017 Jan 7;17:27. doi: 10.1186/s12906-016-1517-y (PMC5219761; doi:10.1186/s12906-016-1517-y)
Supplement: Additional file 1: — NMR spectra of compound. (DOCX 696 kb) [file 12906_2016_1517_MOESM1_ESM.docx]

Various constituents were isolated and they were given various codes, this compound was named as ISM-7. Its spectroscopic data is as:

**
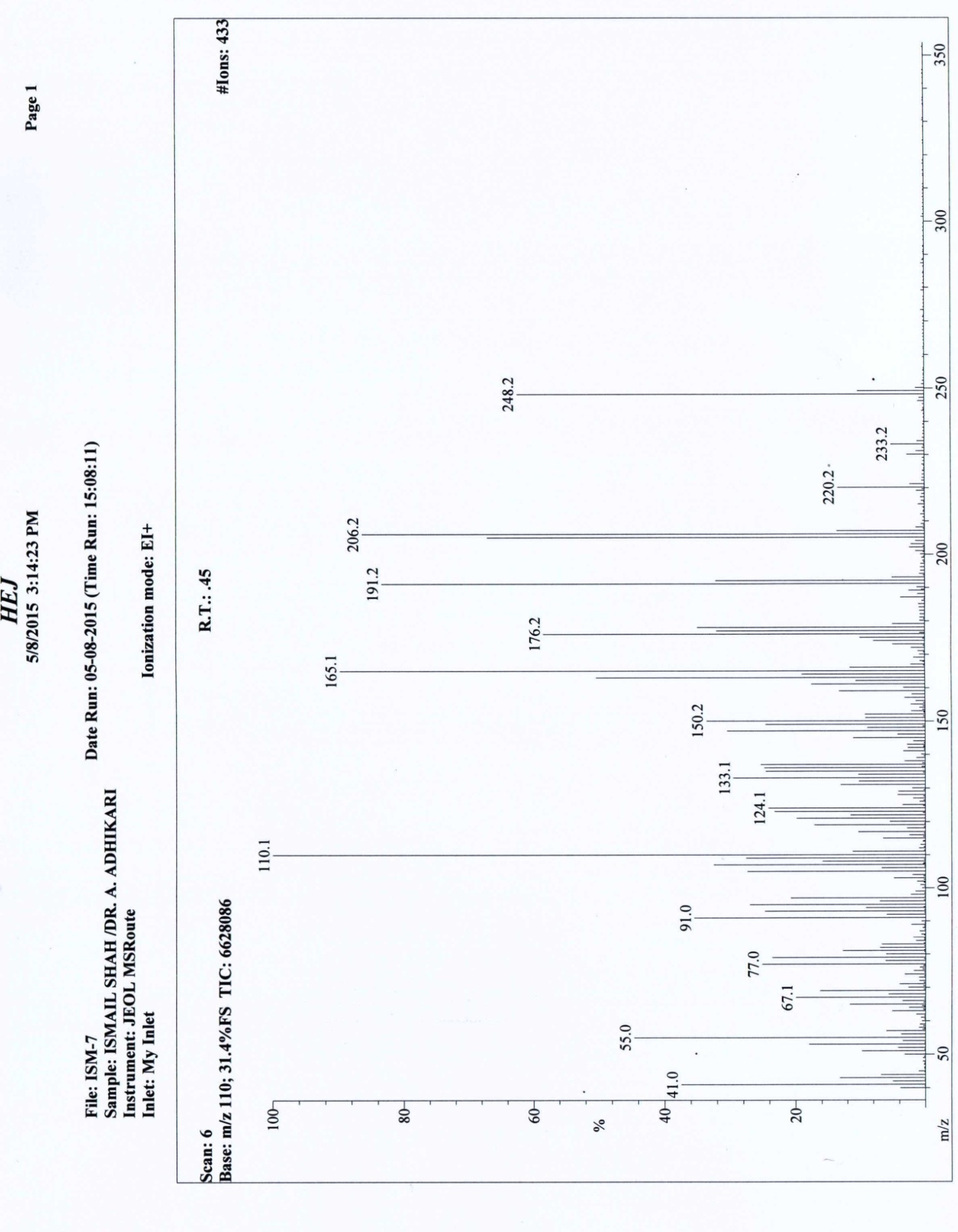
**

**
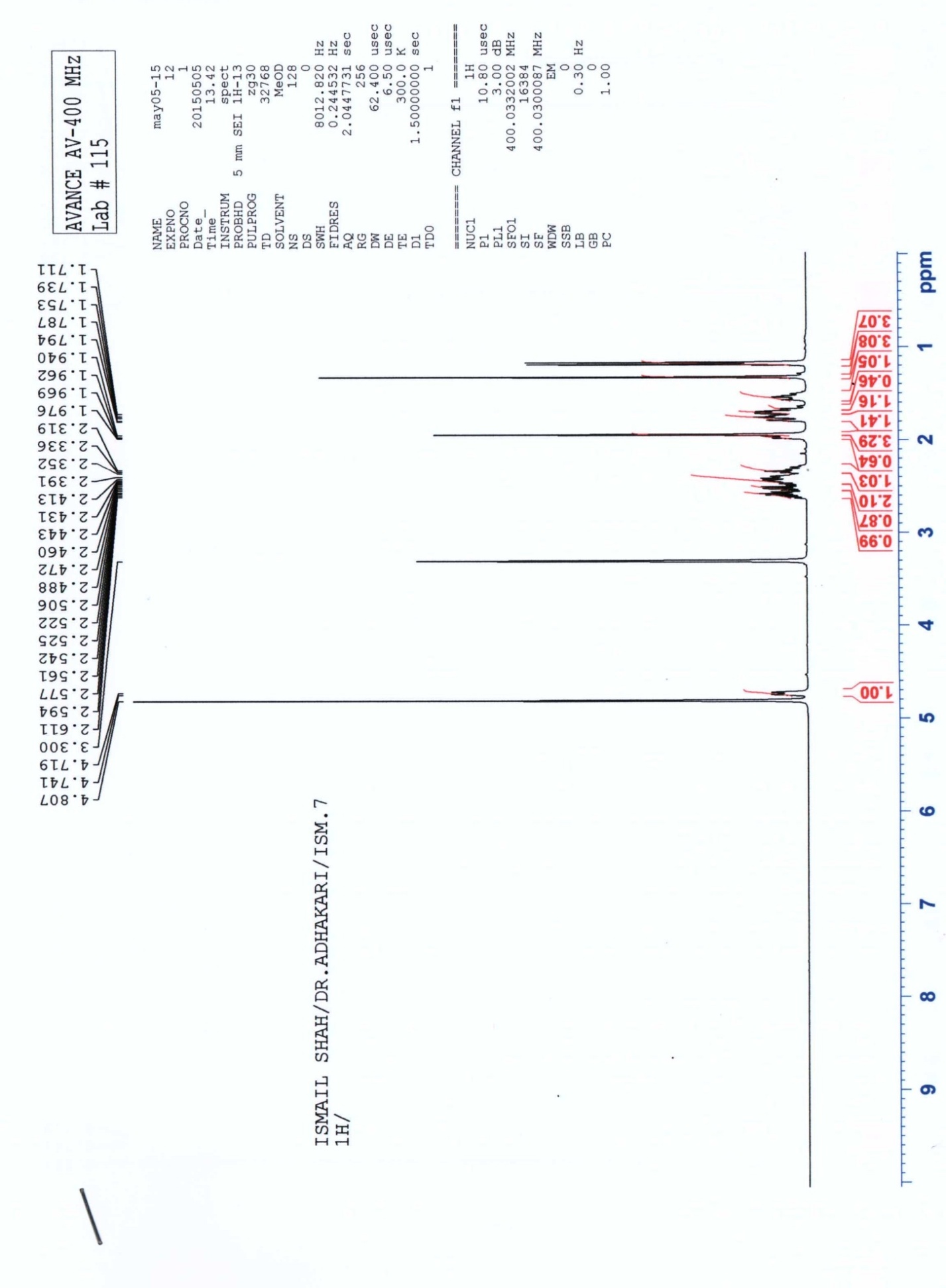
**

**
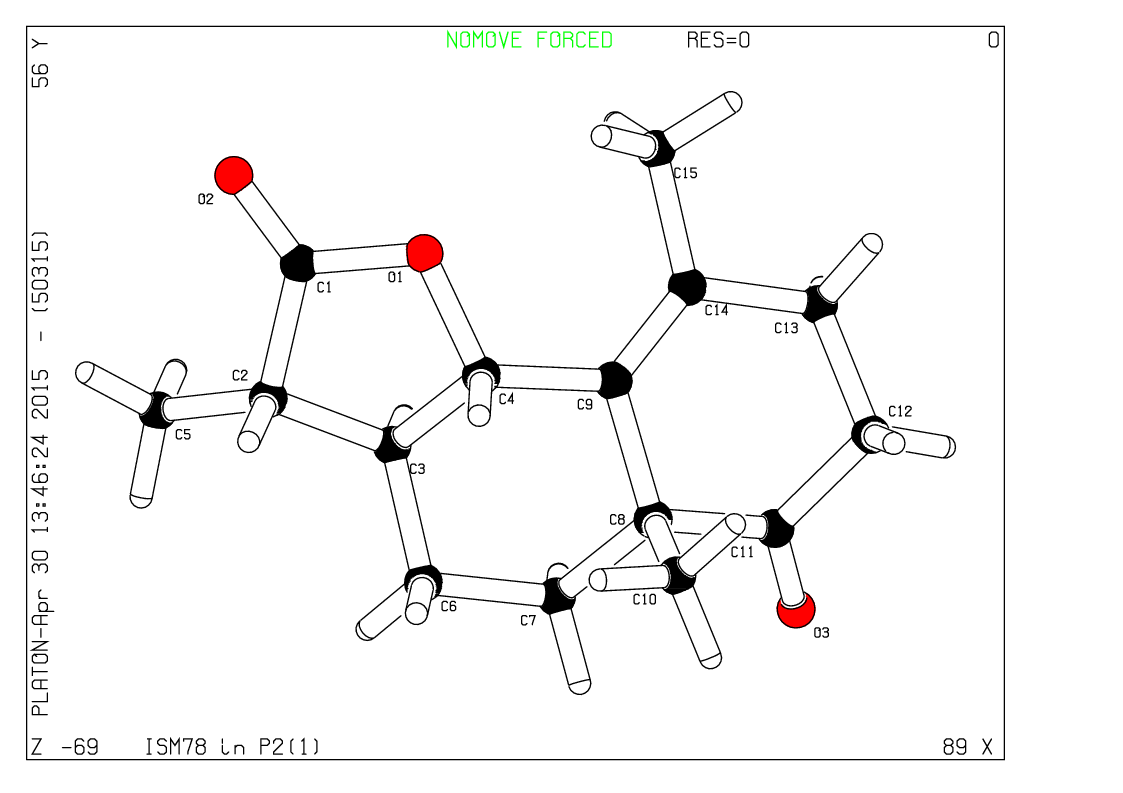
**
